# Associations between Brain Reserve Proxies and Clinical Progression in Alzheimer’s Disease Dementia

**DOI:** 10.3390/ijerph182212159

**Published:** 2021-11-19

**Authors:** Hyung-Jun Yoon, Seung-Gon Kim, Sang Hoon Kim, Jong Inn Woo, Eun Hyun Seo, For the Alzheimer’s Disease Neuroimaing Initiative

**Affiliations:** 1 Department of Neuropsychiatry, College of Medicine, Chosun University/Chosun University Hospital, Gwangju 61452, Korea; YoonHyungJun@chosun.ac.kr (H.-J.Y.); sgkim@chosun.ac.kr (S.-G.K.); shckim@chosun.ac.kr (S.H.K.); 2 Department of Neuropsychiatry, College of Medicine, Seoul National University Hospital, Seoul 03080, Korea; jiwoomd@snu.ac.kr; 3 Premedical Science, College of Medicine, Chosun University, Gwangju 61452, Korea

**Keywords:** Alzheimer’s disease, dementia, cognitive reserve, brain reserve, clinical progression, cerebral glucose metabolism

## Abstract

The purpose of this study was to investigate whether brain and cognitive reserves were associated with the clinical progression of AD dementia. We included participants with AD dementia from the Alzheimer’s Disease Neuroimaging Initiative, provided they were followed up at least once, and candidate proxies for cognitive (education for early-life reserve and Adult Reading Test for late-life reserve) or brain reserve (intracranial volume [ICV] for early-life reserve and the composite value of [^18^F] fluorodeoxyglucose positron emission tomography regions of interest (FDG-ROIs) for late-life reserve) were available. The final analysis included 120 participants. Cox proportional hazards model revealed that FDG-ROIs were the only significant predictor of clinical progression. Subgroup analysis revealed a significant association between FDG-ROIs and clinical progression only in the larger ICV group (HR = 0.388, *p* = 0.028, 95% CI 0.167–0.902). Our preliminary findings suggest that relatively preserved cerebral glucose metabolism might delay further clinical progression in AD dementia, particularly in the greater ICV group. In addition to ICV, cerebral glucose metabolism could play an important role as a late-life brain reserve in the process of neurodegeneration. Distinguishing between early- and late-life reserves, and considering both proxies simultaneously, would provide a wider range of factors associated with the prognosis of AD dementia.

## 1. Introduction

As the global elderly population increases rapidly, the prevalence of dementia is increasing worldwide. In particular, the average prevalence of Alzheimer’s disease (AD) dementia, which is the most frequent cause of dementia, was 14.5% [1]. Given that dementia management is becoming a key public health priority, it is important to determine the prognosis of clinical progression after dementia diagnosis. According to the fifth edition of the American Psychiatric Association’s Diagnostic and Statistical Manual (DSM-Ⅴ) [2], significant cognitive impairment is required to be diagnosed with major neurocognitive disorder, which corresponds to dementia. Cognitive function gradually worsens as dementia advances. In terms of individual perspective, however, there is a large variance in the pattern of clinical progression; for example, some people show greater cognitive decline, but others show less cognitive change during the disease course in dementia. Understanding the reason for such individual differences would constitute an important step toward designing strategies for dementia management and care.

Reserve theory was introduced to explain this individual difference [3]. Some people can tolerate the disease pathology burden better than others; the hypothetical concept of the reserve, against brain pathology, comes from the observation of the discrepancy between brain pathology and clinical manifestation of that pathology. There are two concepts in the reserve model: brain and cognitive reserves. Initially, the differences in brain size and other quantitative aspects of the brain (e.g., the number of neurons or synapses, etc.) were focused to explain the interindividual differences in maintaining cognitive function in the presence of brain pathology. This is called the brain reserve model [4,5,6]. In fact, groups with larger and heavier brains, larger number of neurons, and more functional brain tissue remaining showed less clinical expression of AD dementia [4,5]. The brain reserve can be both static or dynamic; human brains stop growing in the mid-twenties [7], and the attained maximum brain volume at that age is, thus, referred to as the ‘static’ brain reserve [8]. The ‘dynamic’ brain reserve could change, as a function of aging and accumulation of pathology [9], which could lead to the brain becoming functionally adapted to the disease process. On the other hand, the cognitive reserve is an active model that refers to the ability to make flexible and efficient use of available brain reserve when performing tasks [10]. It seems that cognitive reserve supports coping with brain pathology by using compensatory mechanisms [11]. Similar to brain reserves, there are static and dynamic cognitive reserves. Education is considered a static cognitive reserve, because it is generally attained, at a maximum level, in early-life [12]. Cognitive reserve can also be dynamic, because it can change based on life experience [10]. Brain and cognitive reserves are not mutually exclusive. Reserve theory suggests that greater brain reserve supports functional adaptation to clinical progression in neurodegeneration, allowing a greater cognitive reserve [13]. Therefore, each individual’s level of brain and cognitive reserve might be partly responsible for the interindividual heterogeneity in the pattern of clinical progression in AD dementia.

Because reserve theory is a hypothetical concept, it cannot be directly measured. Instead, various measurements have been suggested as a proxy to capture the characteristics of the reserve. Static and dynamic reserves can be captured by early- and late-life proxies, respectively [12]. For example, education is the most typical early-life proxy for cognitive reserve [14,15]. Late-life proxies for cognitive reserve are related to an individual’s attained cognitive ability to respond to environmental stimuli throughout the entire life course [12,16]. Socio-behavioral proxies, such as occupational complexity, level of physical or leisure activities, and test measures for intelligence, are commonly used. More recently, neuroimaging proxies, such as intracranial volume (ICV), specific patterns of gray matter volume, white matter microstructural properties, and PET or fMRI measurement of the functional brain, have been applied to capture brain reserve [10]. ICV may reflect the maximum attained brain size and is considered the most typical early-life proxy of brain reserves [13]. The amount of functional brain tissue remaining at the age of dementia can be a late-life proxy for brain reserve. However, the optimal proxy that best describes the brain or cognitive reserve remains unclear. Identification of both comprehensive and sensitive proxy measures could become a research priority in the field of reserve theory and dementia research.

Brain and cognitive reserves have been extensively investigated in dementia research, especially in AD dementia [12,17,18,19,20,21,22,23]. However, most studies have focused on the relationship between cognitive reserve and the risk of dementia in nondemented older adults. Consequently, there is very limited literature on the relationship between reserve and clinical progression in individuals with AD dementia. A few studies demonstrated that brain and cognitive reserve modified the clinical expression of symptoms in AD [8,24]. Although AD dementia is a progressive disease, brain and cognitive reserve may play a protective or, at least, buffering role for clinical deterioration, even after the diagnosis of dementia. Brain reserve and cognitive reserve are conceptually distinct, but they may be empirically related to each other. However, most previous studies have investigated either brain reserves or cognitive reserves or used only one of the early- or late-life proxies. It is unclear whether these factors independently or interactively influence the clinical progression of AD dementia. Given that brain and cognitive reserves can be changed based on life-span developmental experiences [10], a more comprehensive approach using both brain and cognitive reserves with early- and late-life proxies is, thus, needed.

Therefore, in the present study, we examined whether early- and late-life proxies, for brain and cognitive reserves at baseline, were associated with further the clinical progression of AD dementia.

## 2. Materials and Methods

### 2.1. Participants

This study used data from the Alzheimer’s Disease Neuroimaging Initiative (ADNI) database (adni.loni.usc.edu). Data were downloaded from the website in December, 2020. The ADNI was launched in 2003 as a public-private partnership, led by Principal Investigator Michael W. Weiner, MD. The primary goal of ADNI has been to test whether serial magnetic resonance imaging (MRI), positron emission tomography (PET), other biological markers, and clinical and neuropsychological assessment can be combined to measure the progression of mild cognitive impairment (MCI) and early Alzheimer’s disease (AD). For up-to-date information, see www.adni-info.org. We included participants from the ADNI 1 and 2 phases and who were diagnosed with AD dementia at baseline examination. Detailed eligibility criteria for the ADNI study group have been described elsewhere [25]. Briefly, AD dementia subjects met the National Institute of Neurological and Communicative Diseases and Stroke/Alzheimer’s Disease and Related Disorders Association (NINCDS-ADRDA) criteria for probable AD [26]; all of them had a clinical dementia rating (CDR) ranged 0.5 to 2.0 and MMSE scores between 20 and 26. Participants with any significant neurological disease, other than suspected incipient AD, such as Parkinson’s disease, multi-infarct dementia, Huntington’s disease, normal pressure hydrocephalus, brain tumor, progressive supranuclear palsy, seizure disorders, subdural hematoma, multiple sclerosis, or a history of significant head trauma, were excluded.

We included participants only if they were followed up at least once, and baseline neuroimaging scans were conducted within 3 months of a clinical and cognitive assessment visit. In addition, we included only those with CDR scores and for whom candidate proxies for cognitive or brain reserve were available. The final analysis included 120 patients with AD dementia in the current study.

### 2.2. Clinical Assessment and Cognitive Reserve Proxies

We selected the CDR sum of boxes (CDR SB) score as a clinical status measure with possible scores ranging from 0 to 18. CDR is a widely used measurement for staging clinical severity. Clinical progression was measured as the change scores of the CDR SB between the baseline and the last follow-up. The Functional Activities Questionnaire (FAQ) was used for everyday functioning. It is useful for monitoring functional changes. The FAQ provides a score range is from 0 to 30, with higher scores indicating worse daily functioning [27]. Information on *APOE* genotypes was collected. *APOE* genotyping was performed at the time of participant enrollment in the ADNI study. The methods for determining *APOE* genotypes have been described previously [28]. Briefly, APOE genotypes were determined using standard polymerase chain reaction-restriction fragment length polymorphism. Participants with one or two copies of allele 4 were classified as APOE ε4 carriers (ε+); participants without allele 4 were classified as APOE ε4 non-carriers (ε−).

We further selected cognitive measures from the ADNI participants. We included the American National Adult Reading Test (ANART) [29]. ANART consists of 50 irregular words, and the number of mispronounced words is recorded. The ANART error score (ANARTERR) is generally used for estimating premorbid intelligence [30], because the reading skill is thought to be relatively preserved until the later stages of AD dementia [29]. Global cognitive function was assessed using the Mini-Mental Status Examination (MMSE) score; education was selected as an early-life proxy for cognitive reserve. ANARTERR was selected as a late-life proxy for cognitive reserve, because ANARTEER, at the time of baseline examination, reflects the attained level of verbal intelligence as participants age.

### 2.3. Neuroimaging Data and Brain Reserve Proxies

#### 2.3.1. MRI

We downloaded the processed MRI data from the ADNI. A detailed description of MRI acquisition and processing can be found in a previous report [31]. The volumes of cortical and subcortical structures were segmented using FreeSurfer (http://surfer.nmr.mgh.harvard.edu/). The technical details of the automated reconstruction protocol have been previously described [32,33]. We selected ICV as an early-life proxy for brain reserve. Maximum ICV is generally achieved by puberty and stays constant during adulthood [34,35]. ICV was not affected by the initial AD process [8]. In addition, the medial temporal lobe (MTL) volume, including the hippocampus (HC) and entorhinal cortex (EC), were downloaded. They were selected because the MTL regions are vulnerable to the initial AD pathology process [36].

#### 2.3.2. FDG-PET

For the late-life proxy for brain reserve, we downloaded [^18^F] fluorodeoxyglucose -PET (FDG-PET) data from ADNI. Details of the FDG-PET data acquisition protocol in ADNI are publicly available on the UCLA Laboratory of Neuroimaging (LONI) website (http://www.loni.ucla.edu/ADNI/Data/ADNI_Data.shtml). We selected the composite value of FDG-PET regions of interest (FDG-ROIs) as the cerebral glucose metabolism index in the current study. The ROIs included the bilateral inferior temporal, lateral parietal, and bilateral posterior cingulate cortex regions. These ROIs were generated based on the regions frequently cited in the AD and MCI research and validated for clinical usefulness [37]. The composite value of FDG-ROIs served as a late-life proxy for brain reserve, because it could reflect each participant’s functional level of the brain remaining at the time of baseline examination.

#### 2.3.3. Florbetapir PET

A detailed description of florbetapir PET acquisition and processing can be found on the ADNI website (http://adni.loni.usc.edu/wp-content/uploads/2010/05/ADNI2_PET_Tech_Manual_014201.pdf) or in previously published reports [37]. We collected the mean florbetapir standard uptake value ratio (SUVR) for each participant. Participants were designated as amyloid beta burden-positive (Aβ+) or amyloid beta burden-negative (Aβ−, based on an SUVR cutoff of 1.11 [37].

### 2.4. Statistical Analysis

Baseline demographic and clinical data were compared between the progression and stable groups using separate one-way analysis of variance (ANOVA) and *χ*^2^ test for continuous and categorical variables, respectively. Proxies for brain and cognitive reserve were also compared between groups using separate one-way ANOVAs. Cox proportional hazards models, with the forward conditional method, were conducted to investigate the associations between reserve proxies and further clinical progression. Cox models were adjusted for age, gender, MMSE, and FAQ scores. Further clinical progression (CDR SB change score < 0) was event (coded as 0). The reference group (coded as 0) was the lower reserve group, based on the median value of education, ANARTERR, FDG-ROIs, and ICV. These analyses were performed using SPSS version 26.0 for Windows (SPSS Inc., Chicago, IL, USA); *p*-values less than 0.05 were considered significant.

## 3. Results

### 3.1. Participant Characteristics

We included 120 patients with AD dementia at baseline. Fifty-three (44.2%) were in the very mild stage (CDR 0.5), 66 (55.0%) were in the mild stage (CRD 1.0), and one was in the moderate stage (CDR 1). All of them were followed up at least once. The demographic and clinical characteristics of the participants are shown in Table 1. The mean follow-up length was 11.42 months (SD = 5.82, range 6~24 months). CDR SB change between baseline, and the last follow-ups were ranged from −9.0 (decline) to 2.5 (improve). Based on a CDR SB change score of 0, 87 (72.5%) were identified as the progressive group (ranged from −9.0 to −0.5), while 33 (27.5%) were in the stable group (ranged from 0 to 2.5). At baseline, there were no significant group differences in age, education, gender distribution, or mean follow-up month.

### 3.2. Clinical Characteristics and Reserve Proxies

The progressive and stable groups showed similar levels of clinical severity at baseline, as measured by CDR and CDR SB. In addition, there was no significant group difference, in terms of the mean level of CDR, with values of 0.799 and 0.758 for progressive and stable groups, respectively (*p* = 0.46). Distributions of APOE ε+, Aβ +, and mean MTL volumes (HC and EC) did not differ between the groups. However, the progression group showed significantly lower performance on the MMSE and FAQ scores than the stable group (Table 1). However, when age, gender, and education were adjusted, significant group differences in MMESE (*p* = 0.085) and FAQ (*p* = 0.071) were diminished.

In terms of reserve proxies, the progressive and stable groups showed no significant difference in early-life reserves. That is, education and ICV did not significantly differ between groups. In contrast, the progressive group showed significantly lower levels of late-life cognitive and brain reserves, served by ANARTERR and FDG-ROIs, respectively, than the stable group (Table 1).

### 3.3. Associaations between Reserve Proxies and Clinical Progression

To investigate the associations between possible proxies for reserve and occurrence of further clinical progression in AD dementia, survival analysis, using the Cox proportional hazards model, was conducted. The model was adjusted for age, gender, MMSE, and FAQ scores. We adjusted the MMSE and FAQ in the model, because they were different between groups at baseline. All possible proxies for reserve, including education, ANARTERR, ICV, and FDG-ROIs, were entered into the model. Based on the median values of education (median = 16), ANARTERR (median = 16), ICV (median = 1,490,000), and FDG-ROIs (median = 5.50), groups were categorized into higher and lower reserve groups. FDG-ROIs was the only significant predictor of further clinical progression in AD dementia (HR = 0.562, *p* = 0.033, 95% CI 0.330–0.955) (Table 2). Education (*p* = 0.493), ANARTERR (*p* = 0.904), and ICV (*p* = 0.228) were excluded in the model, because they were not significantly associated with further clinical progression in the whole group.

**Figure 1:**
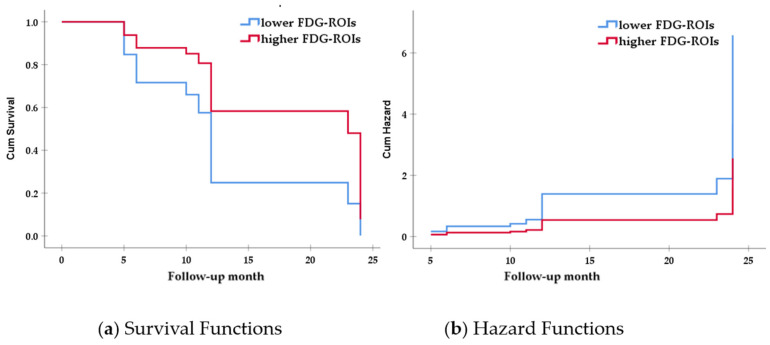
Survival and hazard functions for FDG-ROIs in the larger ICV group. *Note:* (**a**) survival functions; (**b**) hazard functions; red line: higher FDG-ROIs group. Blue line: lower FDG-ROIs group; FDG-ROIs = [^18^F] fluorodeoxyglucose positron emission tomography regions of interest, including the bilateral inferior temporal, lateral parietal, and bilateral posterior cingulate cortex regions.

In addition, we divided our group into smaller and larger ICV groups, based on the median value of ICV, and performed the Cox proportional hazards models separately for each subgroup. A significant association between FDG-ROIs and further clinical progression was observed only in the larger ICV group (HR = 0.388, *p* = 0.028, 95% CI 0.167–0.902). Education (*p* = 0.319) and ANARTERR (*p* = 0.737) were not significantly associated with clinical progression in the larger ICV group. On the other hand, no significant associations between all reserve measures and further clinical progression were found in the smaller ICV group (Table 2, and Figure 1).

## 4. Discussion

It is important to identify factors that have a favorable prognosis for dementia. In the current retrospective study, we demonstrated that brain reserve, not cognitive reserve, was associated with interindividual resilience to further clinical deterioration in AD dementia. Late-life brain reserve, measured by cerebral glucose metabolism, was associated with further clinical progression in AD dementia. Moreover, this association was particularly remarkable in a subgroup with a higher early-life brain reserve (i.e., larger ICV). In contrast, cognitive reserve was not associated with the further clinical progression of AD dementia. Our results could provide theoretical background for strategies to maintain reserve in dementia population. It may help to cope with the negative consequences of AD pathology in dementia patients around the world.

All but one of our study participants had either very mild or mild AD dementia. Although AD dementia has a neurodegenerative process, 15 (12.5%) participants remained the same level of clinical severity, and 18 (15%) participants showed better clinical status during the follow-up period. As expected, the majority of the participants (72.5%) deteriorated clinically. We focused on those participants who did not deteriorate, and explored the factors associated with a buffering role for further clinical deterioration in AD dementia, compared to those with progressive participants. At baseline, the progressive and stable groups did not differ, in terms of demographic characteristics, mean follow-up period, level of clinical severity, genetic risk, Aβ pathology burden, total brain volume, or level of MTL brain atrophy. MTL regions, including HC and EC, could reflect neuropathological staging at the time of the baseline examination. MTL brain atrophy could be at least partially attributable to downstream of tau pathology [38,39], and such brain regions are vulnerable to the initial tau pathology process [36]. Global cognition and daily functional levels were slightly lower in the progressive group than in the stable group, but the significance diminished when demographic variables were adjusted for comparison. These characteristics of study participants indicated that the progressive and stable groups, in the current study, had very similar backgrounds, in general. In particular, given the similar levels of MTL brain atrophy, Aβ pathology burden, and global cognition in the two groups, the progressive and stable groups seemed to be at the same level of AD pathology and cognitive status at baseline. On the other hand, significant group differences were observed only in late-life proxies for cognitive and brain reserve. Our findings may indicate that although both groups had similar levels of pathology and clinical severity of AD dementia, individuals with higher premorbid intelligence and cerebral glucose metabolism were less likely to progress.

More importantly, survival analysis, using the Cox proportional hazard model, revealed that only cerebral glucose metabolism was associated with further clinical progression. We found that the possibility of progression in the group of individuals with higher cerebral glucose metabolism is about 1.8 (1/0.562) times lower than in the group of individuals with lower cerebral glucose metabolism (Table 2). Our preliminary results indicate that a higher late-life brain reserve might provide good prognostic information for the clinical course of AD dementia. Given that FDG-PET is known to be a reliable and sensitive measure of synaptic function [40,41], preserved overall synaptic function in the brain could serve as a valid proxy for late-life brain reserve. Relatively preserved cerebral glucose metabolism might be associated with lifelong cognitive strategies and reflect neuroprotective effect [42]. In line with our results, a longitudinal FDG-PET study on dementia reported that baseline information on regional cerebral glucose metabolism was associated with subsequent disease progression [43]. Besides, the MCI group that did not progress to dementia after 2 years showed higher cerebral glucose metabolism than the MCI group that progressed to dementia [42].

Furthermore, subgroup analysis, based on the size of ICV, revealed that the possibility of progression in the group of individuals with higher cerebral glucose metabolism was about 2.6 (1/0.388) times lower than in the group of individuals with lower cerebral glucose metabolism, only in the larger ICV group (Table 2). The smaller ICV group showed no significant association between reserve measures and clinical progression. Late-life brain reserve might be more strongly associated with clinical progression under conditions of higher early-life brain reserve, as measured by ICV. We speculate that ICV might serve as a moderator variable in the relationship between late-life brain reserve and clinical progression in AD dementia. ICV reflects the absolute limit of individual brain size [13]; however, since some brain areas function independently of brain size, brain size itself does not necessarily capture the entire brain reserve [44]. Moreover, ICV only reflects the static concept of reserve; consequently, it cannot capture the dynamic aspect of brain reserve, influenced by life experience, lifestyle, aging, or pathology. Our preliminary results indicate that more remaining resources of brain function, with larger brain size, may be involved in cognition that allows individuals to maintain their daily cognitive function, in spite of the presence of AD pathology. Both early- and late-life proxies for brain reserve might provide a better prognosis in AD dementia, by promoting brain resilience to neuropathology. The current findings are compatible with the brain reserve theory that modifies the clinical expression of AD pathology [10].

It is also worth mentioning that our results showed that the cognitive reserve played no significant role in buffering disease progression. The progressive group showed lower late-life cognitive reserve, measured by ANARTERR, than the stable group, when using simple comparison (Table 1); however, the significant contribution disappeared in the Cox proportional hazard model (Table 2). This may imply that the influence of late-life cognitive reserve is minor or negligible after the diagnosis of AD dementia. Although brain reserve and cognitive reserve are conceptually distinguishable, they are not clearly distinct entities but, instead, interrelated. Cognitive reserve is the ability to actively cope with the brain pathology, by using brain reserve efficiently [10]. Many previous studies have reported that higher cognitive reserve is associated with a reduced risk of dementia [16,20,22,45,46]. Cognitive reserve might be critical before the onset of dementia. For example, longitudinal studies have demonstrated that higher levels of cognitive reserve are associated with better cognitive performance [47,48] or a reduced risk of dementia development in later life [20,45]. The influence of cognitive reserve on the course of AD-related clinical manifestations might be less clear. One possible interpretation, although speculative, is that once cognitive impairment is clinically apparent and diagnosed with dementia, there would be the least brain resource that cognitive reserve can use efficiently. The brain reserve theory postulates that interindividual differences in susceptibility to brain pathology are a function of a purely quantitative aspect of brain reserve capacity, in addition to the amount of brain damage [11]. Therefore, after a considerable extent of AD pathology to be clinically apparent is reached, quantities of brain structure and function, that is, the brain reserve, would matter. Once diagnosed with dementia, the brain reserve might be a more important contributing factor to resilience to AD-related neurodegeneration than cognitive reserve. Our results suggest that individuals with high brain reserve may preserve their cognitive and daily functioning better than those with lower brain reserve during the clinical course of AD dementia.

Some limitations and directions for future research should be discussed. First, the follow-up period in the current study was relatively short. Since AD-related neurodegeneration worsens over a long period of time, our results are only preliminary and should be interpreted with caution. Longer follow-up for individuals with AD dementia would provide a broader perspective on brain reserve. Second, we could not completely rule out the possibility of the difference in FDG-ROIs, due to the differences in disease pathology burdens. However, we assumed that the progressive and stable groups were in the same level of pathology, because they did not differ in terms of clinical severity, genetic risk, Aβ pathology burden, total brain volume, or level of MTL brain atrophy at baseline. Third, we measured late-life brain reserves using the composite value of cerebral glucose metabolism. That was because of the lack of hypotheses for specific brain regions that are responsible for the reserve theory, as well as the small sample size. Regional information on FDG-PET or a voxel-based approach with a larger sample size would allow for a more detailed description of the role of brain reserve in AD dementia. Lastly, we used one single proxy for each reserve. Future studies are advised to include more proxies for reserve, such as occupational complexity, level of socio-intellectual activities or physical exercise, and other indexes for brain function, in order to capture a wider range of reserves.

## 5. Conclusions

Our preliminary findings suggest that relatively preserved cerebral glucose metabolism might delay further clinical progression in AD dementia, particularly in the greater ICV group. In addition to ICV, which is a widely used proxy for brain reserve, cerebral glucose metabolism could play an important role as a late-life proxy for brain reserve in the process of neurodegeneration. Distinguishing between early- and late-life proxies, and considering both proxies, would simultaneously provide a wider range of factors associated with the prognosis of AD dementia.

## Figures and Tables

**Table 1:** Demographic and clinical characteristics of the participants at baseline.

	Progressive Group (*n* = 87)	Stable Group (*n* = 33)	*F*	*χ* ^2^	*p* Value
Age (SD), y	74.59 (8.07)	73.57 (8.01)	0.38		0.54
^a^ Education (SD), y	15.55 (2.57)	16.21 (2.75)	1.52		0.22
Female, *n* (%)	34 (39.1)	16 (48.5)		0.87	0.35
FU, month	11.68 (5.88)	10.73 (5.69)	0.64		0.43
CDR, 0.5, *n* (*%*)	37 (42.5)	16 (48.5)		0.67	0.72
1.0	49 (56.3)	17 (51.5)			
2.0	1 (1.1)	0 (0.0)			
CDR SB	4.51 (1.67)	4.35 (1.64)	0.23		0.63
APOE ε+, *n* (*%*)	59 (67.8)	23 (69.7)		0.04	0.84
Aβ+, *n* (*%*)	77 (88.5)	27 (81.8)		0.93	0.34
MMSE	22.78 (2.01)	23.67 (2.13)	4.49		0.04
FAQ	13.84 (7.07)	10.91 (6.66)	4.22		0.04
^a^ ANARTERR	17.78 (9.13)	13.94 (9.54)	4.14		0.04
^b^ FDG-ROIs	5.28 (0.68)	5.61 (0.88)	4.78		0.03
HC	5847.26 (852.97)	6008.33 (1079.91)	0.55		0.46
EC	2895.81 (658.73)	3006.46 (678.28)	0.49		0.48
^b^ ICV	1,502,750.00 (172,421.14)	1,527,000.00 (181,148.63)	0.42		0.52

^a^ proxies for cognitive reserve; ^b^ proxies for brain reserve. *Note:* data are presented as means (standard deviations), unless specified otherwise. SD = standard deviation; FU = follow-up; APOE ε+ = apolipoprotein ε4 carriers; Aβ+ = amyloid beta burden positive; CDR = clinical dementia rating; CDR SB = CDR sum of boxes; MMSE = Mini-Mental State Examination; ANARTERR = American National Adult Reading Test error score; FAQ = functional assessment questionnaire; FDG-ROIs = [^18^F] fluorodeoxyglucose positron emission tomography regions of interest, including the bilateral inferior temporal, and lateral parietal, and the bilateral posterior cingulate cortex regions; HC = hippocampus volume; EC = entorhinal cortex; ICV = intracranial volume.

**Table 2:** Results of multivariate Cox proportional hazard model.

	*B*	*SE B*	*Wald*	*p* Value	HR	95% CI	
						Lower	Upper
Total group							
Age	0.018	0.015	1.485	0.223	1.018	0.989	1.049
Gender	−0.098	0.243	0.162	0.688	0.907	0.563	1.460
MMSE	0.014	0.061	0.055	0.815	1.027	0.993	1.062
FAQ	0.027	0.017	2.481	0.115	1.027	0.993	1.062
FDG-ROIs 0: lower * 1: higher	−0.577	0.271	4.532	0.033	0.562	0.330	0.955
Larger ICV group only							
Age	0.004	0.029	0.021	0.884	1.004	0.949	1.062
Gender	0.057	0.554	0.011	0.918	1.059	0.357	3.138
MMSE	−0.062	0.084	0.546	0.460	0.940	0.797	1.108
FAQ	0.035	0.026	1.807	0.179	1.036	0.984	1.091
FDG-ROIs 0: lower * 1: higher	−0.947	0.430	4.838	0.028	0.388	0.167	0.902

* Reference group. *Note:* B = regression coefficient; SE B = standard error of the regression coefficient; Wald = Wald statistic, (B/SE)^2^; HR = hazard ratio; CI = confidential interval; MMSE = Mini-Mental State Examination; FAQ = functional assessment questionnaire; FDG-ROIs = [^18^F] fluorodeoxyglucose positron emission tomography regions of interest, including the bilateral inferior temporal, and lateral parietal, and the bilateral posterior cingulate cortex regions; ICV = intracranial volume.

## Data Availability

This study analyzed data from Alzheimer’s Disease Neuroimaging Initiative (ADNI) database (adni.loni.usc.edu). For up-to-date information, see www.adni-info.org.
